# A global streamflow reanalysis for 1980–2018

**DOI:** 10.1016/j.hydroa.2019.100049

**Published:** 2020-01

**Authors:** Lorenzo Alfieri, Valerio Lorini, Feyera A. Hirpa, Shaun Harrigan, Ervin Zsoter, Christel Prudhomme, Peter Salamon

**Affiliations:** a European Commission, Joint Research Centre (JRC), Ispra, Italy; b School of Geography and Environment, University of Oxford, Oxford, UK; c European Centre for Medium-Range Weather Forecasts (ECMWF), Reading, UK; d Centre for Ecology and Hydrology, Wallingford, UK; e Geography Department, Loughborough University, Loughborough, UK

**Keywords:** Hydrological reanalysis, Global hydrology, Model calibration, Global Flood Awareness System (GloFAS), Distributed modelling

## Abstract

Global and continental scale hydrological reanalysis datasets receive growing attention due to their increasing number of applications, ranging from water resources management, climate change studies, water related hazards and policy support. Until recently, their use was mostly limited to qualitative assessments, due to their coarse spatial and temporal resolution, large uncertainty and bias in the model output, and limited extent of the dataset in space and time. This research reports on the setup of a gridded hydrological model with quasi-global coverage, able to reproduce a seamless 39-year streamflow simulation in all world’s medium to large river basins. The model was calibrated at 1226 river sections with a total drainage area of 51 million km^2^ within 66 countries, using ECMWF’s latest atmospheric reanalysis ERA5. A performance assessment revealed large improvements in reproducing past discharge observations, in comparison to the calibration used in the current operational setup of the hydrological model as part of the Copernicus – Global Flood Awareness System (GloFAS, www.globalfloods.eu), with median scores of Kling-Gupta Efficiency KGE = 0.67 and correlation r = 0.8. The simulation bias was also dramatically reduced and narrowed around zero, with more than 60% of stations showing percent bias within ±20%. Pronounced regional differences in the simulation results remain, pointing out the need for detailed investigation of the hydrological processes in specific regions, including parts of Africa and South Asia. In addition, observed discharges with high data quality is key to achieving skillful model output. The new calibrated model will become part of the operational runs of GloFAS in the next system release foreseen for Spring 2020, together with a near real time extension of the streamflow reanalysis.

## Highlights

• We produced a global daily streamflow reanalysis for 1980–2018.
• The hydrological model is calibrated with observed discharge at 1226 river sections.
• Considerable improvements compared to the operational setup of GloFAS v2.1.
• GloFAS streamflow reanalysis v3.0 is freely available for use in large scale applications.

## 1 Introduction

Knowledge of the hydrological states and their variability in space and time on our planet is key information for a variety of disciplines, including water resources, natural hazards, biodiversity, and energy production. Global hydrological models are effective tools to reconstruct seamlessly the various components of the water balance and reproduce a continuous dataset to be used for further applications. Meteorological datasets, the main dynamic input for hydrological modeling, are increasingly growing in number, quality and spatial coverage. Relevant large scale products are derived from ground observations (e.g., Harris et al., 2014, Haylock et al., 2008, Ntegeka et al., 2013), remote sensing from satellite and ground-based radars, atmospheric reanalysis, and mixed products (see Beck et al., 2019 and references therein). A number of research groups have developed global (e.g., Beck et al., 2017 and references therein; Döll et al., 2003, Fekete et al., 2002, Lin et al., 2019, Qian et al., 2006, Reichle et al., 2011, Sperna Weiland et al., 2010, Yamazaki et al., 2011) and continental scale (e.g., Abbaspour et al., 2015, Alfieri et al., 2014b, Van Dijk et al., 2014, Wongchuig et al., 2019) hydrological reanalysis using various configurations of inputs, models, and parameterization. Among them, several pointed out that relatively large gaps remain between output discharges and observations at gauged river sections, mostly due to quality issues in the meteorological input dataset, incorrect model parameterization, missing or simplified processes, and insufficient space–time resolution of input and output, which often limit model results to qualitative assessments or to selected rivers and regions where the modeling is acceptable. Coordinated multi-model initiatives such as the WaterMIP (Haddeland et al., 2011) and the Earth2Observe (Schellekens et al., 2017) projects aimed to characterize the variability of the simulated runoff in large rivers using ensembles of global hydrological simulations produced at the common grid resolution of 0.5°. Although both projects acknowledged the added value of an ensemble of simulations, results showed large discrepancies due to different concepts and parameterization in modeling the runoff generation (Schellekens et al., 2017) and the lack of a common protocol to calibrate and validate the participating models.

Beck et al. (2017) compared runoff estimates from 10 macro-scale hydrological models with observations from 966 medium-sized catchments around the globe. They found large differences among the different model output, and higher scores for regions with calibrated model parameterization. The calibration always brings benefits in river sections where accurate observations are available, though its effect is reduced as one moves away from the calibration points, even if along the same river network (Xue et al., 2016). As a consequence, the performance of a calibrated hydrological model can vary substantially across different river basins, hence pointing out the importance to calibrate as many river basins as possible. This is often in contrast with the limited availability of observed data, as well as with the considerable computing resources needed to perform each calibration run.

Model calibration is an iterative process involving a large number of model runs where a set of parameters is perturbed, so that differences between the model output and the observations at the corresponding location are minimized. The number of iterations required and the corresponding computing time (proportional to the computing resources) increase with the size of the parameter set to optimize, yet often enabling improved model skills. However, the size of the calibration parameter set should not be too large, to avoid model overfitting and consequent loss of predictive skills outside the calibration period (Wi et al., 2015). Kouchi et al. (2017) tested the sensitivity of calibration parameters to 1) different objective functions and 2) optimization algorithms. They found that most combinations of the two considered sets can achieve skillful results, though resulting in different configurations of the parameter values. Hence, in large scale calibration exercises, the optimal configuration should take into account also the efficiency of the optimization algorithms, that is, favoring those reaching a skillful parameter set with the minimum number of iterations.

In this work we report on the development of a semi-automated calibration procedure of a large scale hydrological model that underpins the Global Flood Awareness System (GloFAS, www.globalfloods.eu, see Alfieri et al., 2013, Hirpa et al., 2018), to improve the simulated output discharge by tuning a set of model parameters. GloFAS is an operational system for global ensemble streamflow modeling, forecasting, and early flood detection, with a forecast horizon up to 30 days and a seasonal outlook up to 4 months ahead. Together with its twin system EFAS, the European Flood Awareness System, GloFAS is an operational component of the Copernicus Emergency Management Service^1^ (CEMS) that provides complementary forecast information to relevant stakeholders and supports flood risk management at national, regional and global level. As of November 2019, the GloFAS-Reanalysis is based on the operational version 2.1, described in details by Harrigan et al. (2019), while this article presents recent research activities that will be included in future system versions.

The calibration tool presented in this work is implemented using a large database of more than 1200 discharge observations worldwide and ERA5 (Hersbach et al., 2018), ECMWF’s fifth generation atmospheric reanalysis dataset, as forcing input. The calibrated model is then rerun to produce a seamless 40-year dataset of daily streamflows with quasi-global coverage. In the operational GloFAS runs, such reference simulation, hereafter referred to as GloFAS-Reanalysis, is updated in near real-time with the latest ERA5 hydro-meteorological input maps as soon as they become available. Similarly to EFAS, the GloFAS-Reanalysis is used with regard to three key aspects: (I) deriving climatological features of river streamflow in each section of the world river network (e.g., average conditions, extremes, flood thresholds, seasonality); (II) creating initial conditions to run hydrological forecasts driven by the latest weather predictions; (III) providing a reference simulation which is as realistic as possible, to be used as a proxy to evaluate streamflow forecasts in every grid point of the simulation domain (Alfieri et al., 2014a). It follows that continuous efforts are dedicated to the improvement of the GloFAS-Reanalysis, to improve the overall quality of GloFAS forecasts as well as their monitoring.

## 2 Material and methods

### 2.1 Data

#### 2.1.1 The ERA5 atmospheric reanalysis

ERA5 is the latest climate reanalysis dataset produced by ECMWF, on behalf of the European Union, through the Copernicus Climate Change Service (C3S) (Hersbach et al., 2018). The first phase from 1979 to the present became available in January 2019, while the second phase extending back to 1950, is planned for release by the end of 2019. ERA5 is openly accessible via the C3S Climate Data Store^2^. It contains estimates of several meteorological variables including air pressure, temperature and wind at different altitudes, precipitation, soil moisture and ocean variables, among others. ERA5 has replaced ECMWF’s previous atmospheric reanalysis ERA-Interim (Dee et al., 2011). Compared with ERA-Interim, ERA5 benefits from 10 years of advances in Numerical Weather Prediction (NWP) (ECMWF IFS Cycle 41r2 (2016) for ERA5, versus Cycle 31r2 (2006) for ERA-Interim). It also uses improved historical observations, boundary conditions, external forcing and has higher spatial (~31 vs ~ 79 km) and vertical (137 vs 60 levels) resolution. In addition, it includes uncertainty information by providing an ensemble of 10 reanalysis members, at a coarser horizontal resolution. The ERA5 dataset includes hourly output, with near-real time availability (2 to 5 day latency) known as ERA5T, which makes it particularly appealing for the daily updating of the global hydrological conditions used to initialize streamflow forecasting systems such as GloFAS. ERA5T is used for the initialization of operational GloFAS forecasts since November 2018. The improvements introduced in ERA5 include features such as tropical cyclones, better orography, coastal process and related weather phenomena, and medium-range atmospheric forecasts, initialized with ERA5, show an increase in skill of about one day in comparison to those initialized with ERA-Interim (Hersbach et al., 2018). Recent studies in the United States have shown that ERA5 performs substantially better than ERA-Interim in modeling precipitation (Beck et al., 2019), as well as when used to force land surface (Albergel et al., 2018) and hydrological (Tarek et al., 2019) models.

In this study we used daily maps of precipitation, daily mean surface air temperature and relative humidity, incoming solar radiation, net longwave radiation and mean wind speed extracted from ERA5 for 40 complete years between 1979 and 2018. This is one of the main changes in comparison with the previous calibration exercise, based on the same variables, yet extracted from the control run of ECMWF reforecasts in the period 1995–2015.

Some variables were processed to compute estimates of potential evapotranspiration using the Penman-Monteith equation as described in Supit at al. (1994). This is one of the main dynamic input variables of the hydrological model Lisflood, together with daily mean surface air temperature and daily precipitation. ERA5 input variables were aggregated from hourly to daily values and downscaled from the original resolution to the output resolution of 0.1 deg through a bilinear interpolation method.

### 2.2 Methods

#### 2.2.1 Hydrological model and calibration parameters

Hydrological simulations are performed with Lisflood (van der Knijff et al., 2010), a distributed semi-physically based model developed at the Joint Research Centre (JRC) of the European Commission. Such model differs from the operational GloFAS setup (i.e., calibration version v.2018), which is based on a combination of the Hydrology - Tiled ECMWF Scheme for Surface Exchanges over Land (HTESSEL, Balsamo et al., 2011), ECMWF’s land surface scheme, and a simplified version of Lisflood to simulate the groundwater processes and the river routing (see Alfieri et al., 2013). The choice of a different modeling framework for the new GloFAS version is motivated by the need for better control on all the calibration parameters through the use of only one model, as well as the need to maintain only one Lisflood version to be used both in EFAS and GloFAS, so that any model development benefits both early warning systems.

Processes simulated by Lisflood include soil freezing, snowmelt, surface runoff, lakes and reservoirs, water abstraction, infiltration, preferential flow, redistribution of soil moisture within the soil profile, drainage to the groundwater system, groundwater storage, and base flow. Surface runoff is produced at every grid cell and routed through the river network using a kinematic wave approach (Chow et al., 1988). The current model version includes 463 lakes and 687 reservoirs (Zajac et al., 2017), selected among the world’s largest ones listed in the Global Lakes and Wetlands Database (GLWD, Lehner and Döll, 2004) and the Global Reservoir and Dam Database (GRanD, Lehner et al., 2011). The lake outflow is related to the lake level using the Poleni weir equation (Bollrich, 1992). For reservoirs, the outflow is calculated with a set of rules depending on their filling level (see Burek et al., 2013, Zajac et al., 2017).

Accurate and up to date spatial information in the hydrological modeling is important to avoid over‐parameterization and reduce the dimensionality of the calibration. Spatial datasets used in Lisflood include topography maps (i.e. digital elevation model, local drainage direction, slope gradient, elevation range), land use (i.e. land use classes, forest fraction, fraction of urban area), soil (i.e. soil texture classes, soil depth), and channel geometry (i.e. channel gradient, roughness coefficient, bankfull channel depth, channel length, bottom width, and side slope). Most input datasets, parameters and variables necessary for the model are estimated a priori from available global products, such as the Shuttle Radar Topography Mission (SRTM, Jarvis et al., 2008) dataset for elevation, the GlobCover 2009 (Bontemps et al., 2011) for land use, the SoilGrids1km database (Hengl et al., 2014) for soil information, the global river network database (Wu et al., 2012) for river network and flow direction, the Global Width Database of Large Rivers (Yamazaki et al., 2014) for river widths, the SPOT-VGT data (http://wdc.dlr.de/data_products/SURFACE/LAI/) for monthly maps of Leaf Area Index (LAI), among others. Water abstraction maps are representative of the year 2000. They are derived from the work by Wada et al. (2011) and modeled with 12 monthly maps for the domestic and livestock sector and with a constant pattern for the energy and industrial sector. Lisflood underpins a number of large scale applications, particularly over Europe, where it is used in the context of the European Flood Awareness System (EFAS, see Alfieri et al., 2014a, Bartholmes et al., 2009, Thielen et al., 2009) and in climate change impact assessment studies (Alfieri et al., 2015, Dankers and Feyen, 2009, Rojas et al., 2012). In this work, we used a quasi-global setup spanning latitudes 60°S to 90°N over a 0.1°grid and 1-day time step, covering all the main Earth’s land areas except Antarctica, Greenland and Iceland.

A set of eight model parameters was selected for calibration (see Table 1) following recommendations from previous works (Beck et al., 2017, Hirpa et al., 2018), who identified the most relevant model parameters for Lisflood in terms of model sensitivity and uncertainty of the default values. Differently from those works, no calibration parameter specific to lakes and reservoirs was included 1) to limit the dimensionality of the optimization process and the overall number of model runs and 2) so that all catchments ultimately have the same number of calibrated parameters (i.e., including those with no lake or reservoirs). The impact of such choice will be investigated in the evaluation phase, particularly for reservoirs, where the actual operation rules may differ from the modelled one. Further details on the modeling of reservoirs in Lisflood are included in the Supplement.

**Table 1:** Calibrated model parameters, lower (min) and upper (max) perturbation range, and initial value before the perturbations (default). The default value is also used for all uncalibrated catchments.

Parameter name	Description [units]	min	max	default
UpperZoneTimeConstant	Time constant for the upper groundwater zone [days]	3	40	10
LowerZoneTimeConstant	Time constant for the lower groundwater zone [days]	40	500	100
GwPercValue	Maximum rate of percolation from the upper to the lower groundwater zone [mm day^−1^]	0.01	2	0.8
GwLoss	Maximum rate of percolation losses from the lower groundwater zone [mm day^−1^]	0	0.5	0
b_Xinanjiang	Power in the infiltration equation based on the Xinanjiang model [-]	0.01	1	0.5
PowerPrefFlow	Power in the preferential flow equation [-]	0.5	8	4
SnowMeltCoef	Degree-day factor controlling the rate of snowmelt [mm °C^−1^ day^−1^]	2.5	6.5	4
CalChanMan1	Multiplier applied to the Manning’s roughness coefficient of the channel system [-]	0.1	15	3

#### 2.2.2 Station selection

River stations selected for calibration stem from a global database of about 2100 stations maintained in-house at the JRC, resulting from the collection of discharge observations from around 30 data providers worldwide. We performed a screening to identify the stations to calibrate, following recommendations from previous calibration exercises. The following procedure was thus implemented:

• Stations where the drainage area was not reported by the provider were initially removed.
• The absolute relative difference between the drainage area derived from the model flow direction map and reported by each data provider had to be smaller than 20% (e.g., Xue et al., 2016)
• The official drainage area had to be larger than 5,000 km^2^ (following the recommendations by Alfieri et al., 2013).
• At least 4 years of observed daily data had to be available (i.e., 4*365 values, excluding gaps) in the calibration period. Remaining data, up to 8 years were used for model validation, while in case of time series longer than 8 years the dataset was split equally between calibration and validation as suggested by Klemeš (1986).
• When two stations fell in the same grid point, the station with the longest observed time series was retained (14 stations were removed by this criterion).
• Some stations which were close to each other along the same river were removed. The idea behind this criterion is to avoid calibrating clusters of stations, where the downstream ones bring little benefit and often generate anomalous calibrated parameter values, as they are constrained by the simulated inflow of the upstream station and by the discharge rating curve at each station and their uncertainty. To this end, we numerically identified the upstream/downstream relation among stations lying in the same river basin. Then, a threshold value of 10% was imposed as minimum relative difference in drainage area of a downstream station to the closest one along the upstream river network, and removed the stations with smaller relative difference.
• A number of stations were then removed or added on the basis of a comparison and skill evaluation between the observed discharge time series and the corresponding model run with default (i.e., uncalibrated) parameters. This step was necessary to remove data pairs with unexplained large differences, possibly related to a combination of wrong station positioning, large human influence, and errors in the metadata, in the reported discharge time series, in unrepresented processes, or in the model input (e.g., Hamilton and Moore, 2012, Wang et al., 2018). Also, it compensates for the absence of a dedicated data quality control system on the observations dataset. As a result, we removed all stations with a ratio larger than 11 between the average observed and simulated discharge time series as well as its inverse ratio (i.e., Pbias < -91% or Pbias greater than 1000%). In addition, stations previously excluded due to drainage area not reported by the provider were reinstated if the KGE in the uncalibrated run was larger than zero, hence proving some modeling skills (Knoben et al., 2019).

**Fig. 1:**
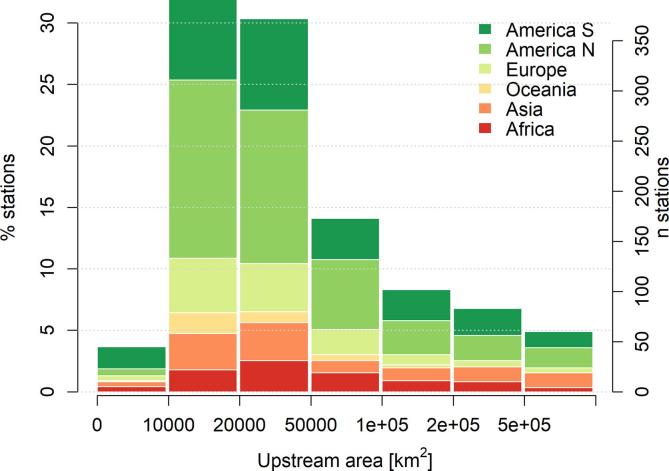
Histogram of the basin upstream area for the 1226 calibration stations, further classified by continent.

**Fig. 2:**
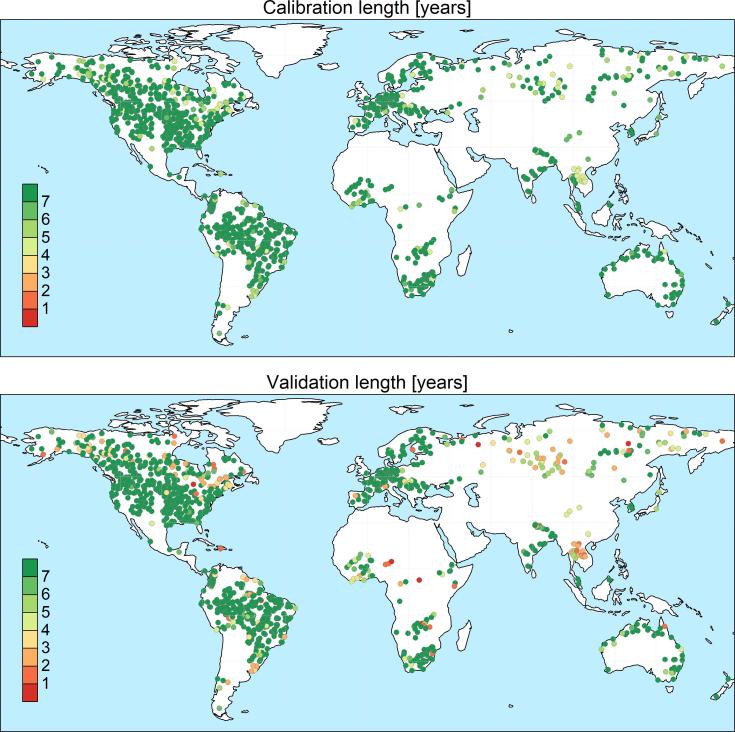
Length in years of the available time series for calibration (top) and for validation (bottom).

The selection procedure described above produced a list of 1226 calibration stations, with a total drainage area of 51 million km^2^, corresponding to 38% of the simulated domain. This results in a mean river basin size of 42,000 km^2^, a size range between 5,000 and 4,680,000 km^2^, and 62% of calibrated river basins having a drainage area ranging between 10,000 and 50,000 km^2^ (see Fig. 1). The spatial distribution of the stations is shown in Fig. 2, together with the respective length of the calibration and validation period.

#### 2.2.3 Calibration procedure

The calibration exercise was performed through perturbation of the eight chosen parameters of the Lisflood hydrological model within realistic ranges (see Table 1) to maximize the skills in the simulated discharge, compared to the corresponding observed time series in the matching river station. The tool used for the hydrological model calibration consists of a set of scripts written in Python and developed in-house at the JRC. The version used in this work is an update of earlier versions (from Beck et al., 2016, Hirpa et al., 2018) focusing on creating a semi-automated tool able to run unsupervised and calibrate in sequence all selected catchments. We used a multisite cascading calibration (MSCC) approach (Xue et al., 2016), where the calibrated discharge from upstream river basins is used as input for downstream ones. The former MS Windows based version was modified for running in a High Performance Computing (HPC) Linux cluster. The implemented version performs the following steps:

a. Filtering of stations on the basis of the length of the available data in the simulation period and optimal selection of calibration and validation period.
b. Hierarchical classification of the calibration stations along the river network and filtering of stations according to the criteria of proximity (see Sect 2.2.2).
c. Subdivision of the stations into a user-defined number of lists, to be run in parallel each on a different cluster node. Every list retains hydrographic consistency, so that all stations within the same river basin fall in the same list.
d. Preparation of input and static data for each catchment to calibrate. All maps are cut out from the global domain in order to reduce the required storage and maximize the speed of execution. Inflows from upstream calibrated catchments are imported, when existing.
e. Run of the simulations and parameter calibration, until user-defined improvement criteria are met. To avoid unnecessary model runs, the calibration tool evaluates the objective function (i.e., KGE) at each generation from the 6th onwards and terminates the calibration process if the improvement compared to the previous generation is smaller than 0.001. The maximum number of generations was limited at ng_MAX_ = 16.
f. When all catchments are calibrated, the routine produces global parameter maps, figures and tables of skill scores for evaluation and diagnostic, and performs data cleaning.

**Fig. 3:**
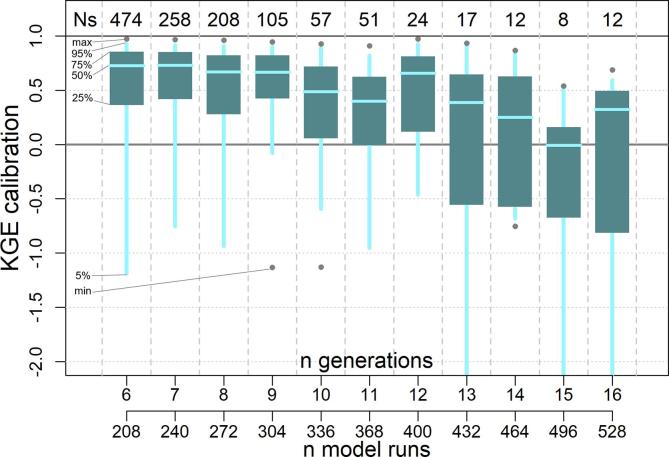
Box plot of the calibration KGE per number of model runs. Ns indicates the number of stations for each bin.

To perturb the calibration parameters we used the (μ + λ) evolutionary algorithm implemented in the Distributed Evolutionary Algorithms in Python (DEAP) toolkit (Fortin et al., 2012). The population size (μ) was set to 16 and the recombination pool size (λ) to 32, thus generating a maximum number of model runs per sub-catchment of μ + λ ng_MAX_ = 528 (see Fig. 3). Each generation produces λ offspring, which are evaluated when the population of the next generation is selected from both offspring and population. Crossover and mutation probabilities were set to 0.6 and 0.4, respectively, which we found to be an effective combination in terms of speed of convergence and avoidance of local minima. Each model run covers seamlessly all timesteps included in the calibration period, including gaps in the time series of observed discharge data. In addition, each model run is initialized one year before the start of the calibration period, to enable the model to warm-up before evaluating its skill against the observations. This was necessary to compensate for the large uncertainty in the initial model states. Model runs are composed of two simulations: a pre-run, to estimate maps of average inflow to the lower groundwater zone and of average discharge, which are used in a subsequent run to compare simulated with observed discharges.

#### 2.2.4 Performance metrics

We used a set of performance metrics to evaluate the model skills in representing observed discharges in the simulation period. The Kling-Gupta efficiency (KGE, Gupta et al., 2009) was used both as objective function during the calibration and as performance metric to compare station results. It is defined as:

$$\mathrm{KGE}=1-\sqrt{{\left(r-1\right)}^{2}+{\left(\beta -1\right)}^{2}+{\left(\alpha -1\right)}^{2}}$$

where *r* is the Pearson product-moment correlation coefficient, β indicates the bias between observed and simulated flows, and α is the variability ratio between observed and simulated standard deviations of the flow.

Similarly to the Nash-Sutcliffe efficiency (NSE, Nash and Sutcliffe, 1970), the *KGE* provides a dimensionless decomposition of the mean squared error (*MSE*) by explicitly accounting for correlation, bias and variability of the flow. To complement the model diagnostic and skill assessment, the *NSE*, the Pearson correlation coefficient (*r*), and the percent bias (*Pbias*) were also analyzed individually. The use of dimensionless metrics is key for large scale and global applications, so that river sections with different climate, flow regime and magnitude can be compared in the same analyses.

## 3 Results

**Fig. 4:**
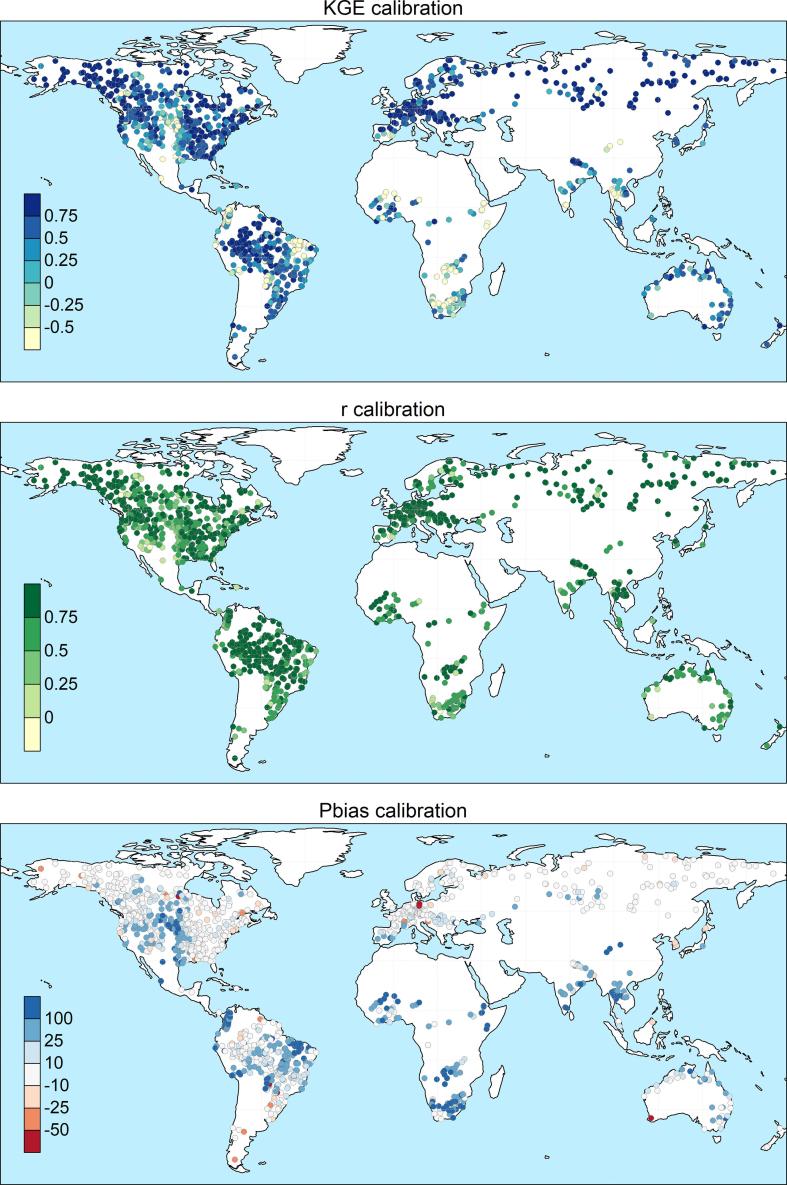
Calibration performance: KGE (top), correlation (center), and percent bias (bottom).

**Fig. 5:**
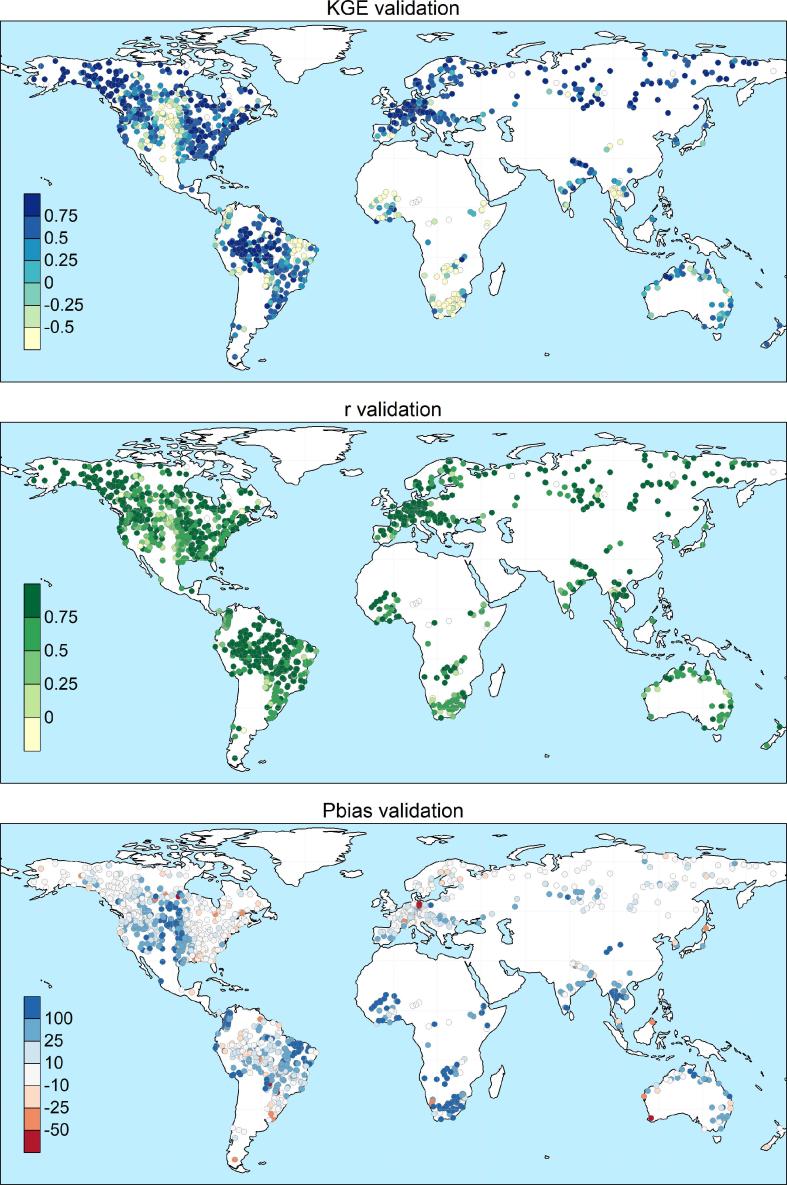
Validation performance: KGE (top), correlation (center), and percent bias (bottom).

**Fig. 6:**
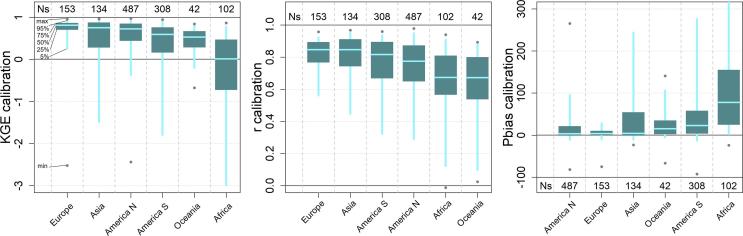
Box plot of the calibration performance by continent: KGE (left), correlation (center), and percent bias (right). Ns indicates the number of stations for each bin. For each performance score, bins are ranked left to right from the most to the least skillful, according to the median of each bin.

**Fig. 7:**
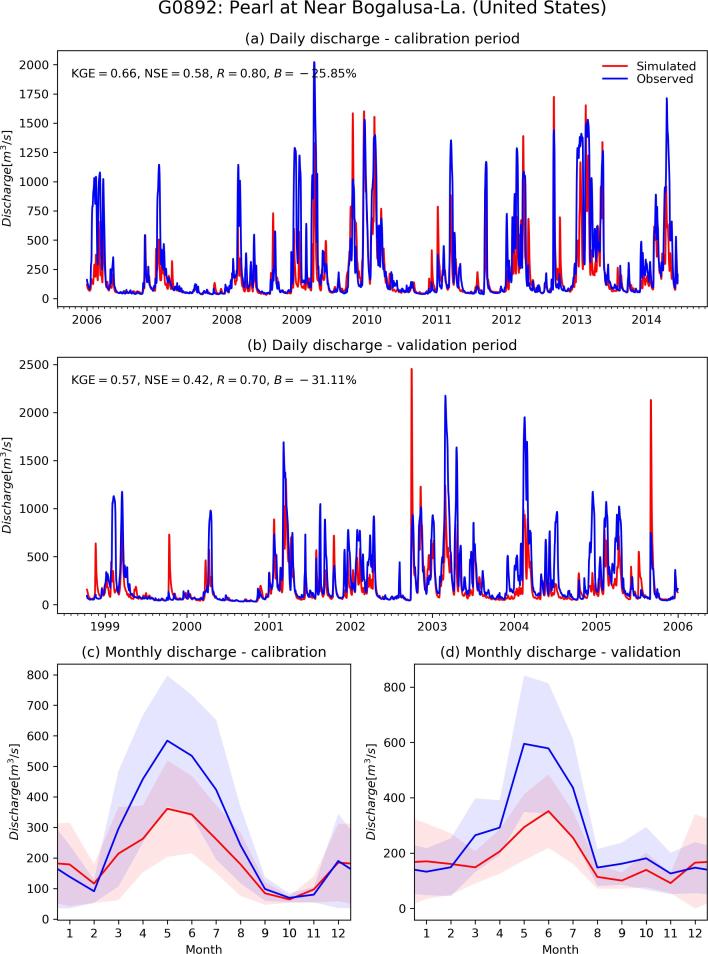
Comparison of simulated and observed discharge for a sample station in the USA, with basin area of about 17,000 km^2^. (a) Calibration period, (b) validation period, seasonality in (c) calibration and (d) validation. Color shading indicates ± 1 standard deviation of the data.

The calibration exercise took overall 2 months to complete on a Intel Xeon HPC cluster, using 10 nodes with CPU E5-4620 @ 2.20 GHz. Each node had 32 cores each, which corresponds to the recombination pool size (λ), meaning that at any given time, 10 sub-catchments could run all 32 simulations of the same generation. A total of 320,000 model runs (640,000 including the pre-runs) was performed to calibrate the 1226 stations, hence an average of 261 runs per station. In 85% of cases (i.e., 1045 out of 1226) the calibration ended within 9 generations, corresponding to 304 model runs (Fig. 3). Despite the additional model runs, calibration performance are on average lower for the remaining stations, suggesting that causes are to be sought in the mismatch between model and observations, rather than in the search algorithm. The geographic distribution of skill scores comparing simulated and observed discharges in calibration and validation is shown in Figs. 4 and 5 respectively. As expected, performance are higher in calibration than in validation, with highest KGE in the northern mid-latitudes and in most of South America. Overestimation of discharges (i.e., Pbias greater than 0) causes reduction of the modeling skills in some areas in the central USA, Brazil, Colombia, South-East Asia and most stations in Africa. Negative bias is less frequent and very location specific, suggesting the effect of human influence and possible issues with the observations and their geolocation on the modeled river network. Pooling results by continent confirms the poorer performance in Africa (median KGE = 0.02), especially due to large positive bias, and to some extent in Oceania (median KGE = 0.53), due to limited correlation values (Fig. 6). Further analysis related the calibration skills to the average specific discharge, to investigate trends over dry or wet regions. Interestingly, the KGE and correlation versus specific discharge take on a U-shape, with highest skills at the two side bins and poorest in the central bin (see Supplement Figure S1). However, differences among classes were not statistically significant in a Kruskal-Wallis rank sum test (Kruskal and Wallis, 1952). Also, the calibration performance shows considerable differences when grouped by provider of discharge observations (Supplement Figure S2 and S3), suggesting the need to further investigate quality control methods of collected hydrological data. Yet, most station data were collected through the Global Runoff Data Centre (GRDC), while several other data providers are associated with a small number of stations, usually in the same area, where the hydrological simulations may suffer from a common source of error (Figure S2). This is partly due to the spatial distribution and the density of data sources used to produce the meteorological forcing and the background maps used in the hydrological simulations, which are abundant in some regions while they are sparse in others (e.g., Berry et al., 2007, Hengl et al., 2014). Overall, median scores in calibration (validation) are KGE = 0.67 (0.61), r = 0.8 (0.78), NSE = 0.42 (0.35) and PBias = 8% (15%). Fig. 7 gives a graphical example of observed versus simulated discharge time series in calibration and validation, for one calibration point with KGE and correlation close to the respective medians (i.e., the 50^th^ percentile) of the entire stations set.

**Fig. 8:**
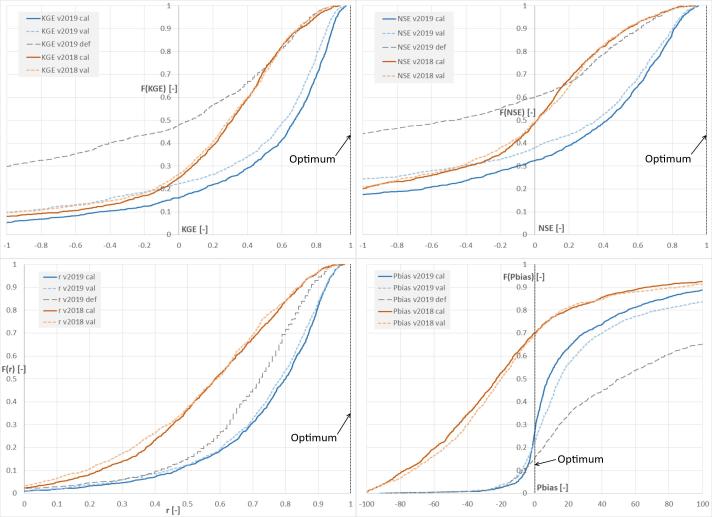
Cumulative distribution of the model performance at the calibrated stations including KGE (top-left), NSE (top-right), correlation (bottom-left), and percent bias (bottom right). Lines refer to the 2018 calibration exercise (in brown) and to this study (blue and grey lines). Grey lines represent performance of the default (def) setup before calibration. Black dotted lines show the case of perfect match between simulations and observations. (For interpretation of the references to color in this figure legend, the reader is referred to the web version of this article.)

In Fig. 8, the cumulative distribution F(·) of the four performance scores in calibration and validation resulting from this work (in the figure referred to as “v2019”) are compared with those of the previous model calibration carried out in 2018 (“v2018”, see Hirpa et al., 2018), and with those of the uncalibrated setup. The KGE curve of the calibrated stations shows a significant improvement compared to both the uncalibrated run and to the previous calibration work. By defining as “skillful” those stations with positive KGE (KGE + ), the ideal case where all stations have perfect calibration skill (i.e., KGE = 1) would generate an area (A) under the positive side of the KGE curve (i.e., the integral of KGE + over the y axis) of *A_KGE+_opt_* = 1 (where “opt” stands for optimum). Results from the calibration (cal) work produced *A_KGE+_v2019 cal_* = 0.56 which results in over twice the value of the uncalibrated (def) run *A_KGE+_v2019 def_* = 0.25 and significantly larger than the previous calibration work *A_KGE+_v2018 cal_* = 0.32. Interestingly, the correlation curve of the uncalibrated run is entirely more skillful than that of the calibration work v2018, hence supporting the choice of the new model setup used in this work. The cumulative distribution of the Pbias indicates substantial differences between the new and the previous calibration setup, where Hirpa et al. (2018) reported large underestimation errors that could not be compensated with the model calibration. Differently, results of the current calibration indicate positive bias in 74% of stations, yet with a narrowing of the distribution of Pbias values around zero. This can be partly attributed to the change in meteorological forcing used in the calibration, from the control run of ECMWF reforecasts (1995–2015) in ver. 2018 (i.e., GloFAS v2.1), to the current approach based on ERA5. For comparison, 61% of calibration stations have Pbias within ± 20%, while in the previous calibration work it was true only in 28% of the cases.

**Fig. 9:**
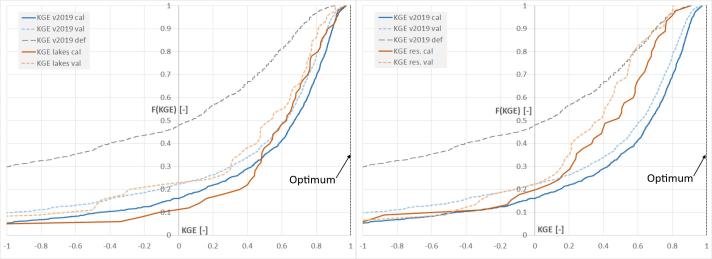
Cumulative distribution of the model KGE resulting from this study for all stations (blue and grey lines), and for stations located at lake outlets (brown lines, left panel) and at reservoir outlets (brown lines, right panel). Grey lines represent performance of the default setup before calibration (all stations). Black dotted lines show the case of perfect match between simulations and observations. (For interpretation of the references to color in this figure legend, the reader is referred to the web version of this article.)

In Fig. 9 the cumulative distribution of the KGE of all calibrated stations is compared with two subsets, including only lake and reservoir outlets, respectively. Performance at 50 lake outlets are in line with those of the full stations set, with an area (*A_KGE+_lakes cal_* = 0.56), thus supporting the choice not to calibrate an additional parameter for lakes, as done in the previous calibration work. On the other hand, performance in stations located downstream of 45 reservoirs (res) are on average poorer (*A_KGE+_res cal_* = 0.41). This reflects the challenges to accurately represent the complex dynamics of water release and diversion from reservoirs, driven by concurrent needs including hydropower, irrigation, flood protection, environmental flows, and recreation activities, among others (Hanasaki et al., 2006, Shen et al., 2012).

## 4 Discussion

Results indicate higher skills in data rich areas, especially in Europe and North America, and in general in the northern mid-latitudes, where the station density is higher. Calibration performance are specific of each river station, though they are not representative of neighboring uncalibrated river basins. Although the model parameters set in the uncalibrated regions were generated by expert judgement, performance outside the calibrated basins are not monitored due to the lack of validation data. Results for the uncalibrated setup in Fig. 8, Fig. 9 (in grey) are representative of the average model performance that one can expect outside the calibrated basins. It is worth noting that the performance in simulating quantitative streamflows are not directly linked to the skills in flood early warning based on threshold exceedance analysis as in GloFAS (Alfieri et al., 2013). Continuous efforts are invested in expanding the coverage and the period of availability of the discharge observation dataset, not only to improve the hydrological modeling skills but to quantify it too. Regionalization techniques of calibrated model parameters have already shown a number of successful applications to improve hydrological modeling in ungauged regions (e.g., Beck et al., 2016, Döll et al., 2003, Nijssen et al., 2001, Samaniego et al., 2010, Widén-Nilsson et al., 2007), though their use in operational systems has been scarce to date.

This work pointed out the need for effective data quality control on discharge time series to use for model calibration. Calibration with poor quality data is to be avoided at all costs, as it can compromise the modeling performance and lead to worse results as compared to the non-calibration case. Poor quality data can manifest themselves in various ways (e.g., Boughton, 2006), and are therefore particularly challenging to spot through automated procedures. In this work, the issue is complicated by the large number of data providers, which increases the likelihood of errors in the data, as well as issues in units conversion and data formatting.

The comparison of the calibration results versus the previous GloFAS setup highlights substantial upgrades, which are likely to improve the general quality of GloFAS forecasts. These improvements are related to different factors of the modelling chain: 1) the use of a different hydrological model, Lisflood, in place of the previous two-model setup to compute land surface fluxes and river routing (see Alfieri et al., 2013). The latter was recognized to be a sub-optimal solution, as the two models are not developed in an integrated way and no feedback between the two is implemented. Also, the previous model calibration was performed only on the routing parameters, so that errors in the land surface scheme were corrected in the wrong place. 2) The new model setup makes use of the ERA5 meteorological reanalysis dataset, based on the ECMWF’s Integrated Forecasting System (IFS) Cycle 41r2. It benefits from a decade of developments in model physics, numerics and data assimilation, compared to the previous reanalysis dataset ERA-Interim (see Sect. 2.1.1), which was used in the previous GloFAS setup. 3) An improved multi step approach to select stations to calibrate, which minimizes incorrect station positioning on the model river network and removes those with poor data quality. The list of calibration stations is the result of a continuous effort to include more data providers, especially in underrepresented regions, and of a more stringent selection criteria. We ultimately generated a list with similar size (i.e., 1226 stations) to that of the previous calibration exercise (i.e., 1287 stations), though with better distribution and data quality. For example, the final list counts 66 world countries with at least one calibration stations, while in the previous version it was only 56.

The substantial and concomitant changes from the previous calibration exercise make it difficult to discern the contribution of specific changes to the overall model improvement. Changes were driven by different rationale, ranging from technological updates, need for model usability and flexibility, data quality issues, among others. Our ultimate goal is to produce the best possible global streamflow reanalysis, which can be extended consistently in near real time and used to initialize medium range and seasonal forecasts in an operational system for streamflow forecasting and flood early detection.

## 5 Conclusions

This article describes the procedure to generate a new global streamflow reanalysis for 1980–2018, with daily time step and 0.1° spatial resolution (~11 km at the Equator), to be used within the Copernicus-EMS Global Flood Awareness System, as well as in a wide range of global modeling frameworks. It is based on the ERA5 meteorological dataset as input and calibrated at 1226 river sections. The GloFAS- Reanalysis dataset v3.0 thus produced is freely available for download through the JRC Data Catalogue (https://data.jrc.ec.europa.eu/collection/id-00288) and near real time updates will be made available through the Copernicus Climate Data Store (https://cds.climate.copernicus.eu) once the operational version is released. The model performance show a substantial improvement of the new calibrated setup in comparison both to the uncalibrated run and to the previous calibrated GloFAS setup. This is likely to improve the operational flood forecasts in GloFAS, through more realistic initial conditions and more consistent warning thresholds. The calibrated model setup will be part of the next major GloFAS upgrade (i.e., GloFAS v3.0), foreseen for early 2020, and will be preceded by an extensive testing and evaluation over a large number of past forecasts.

We are committed to a continuous improvement of the GloFAS-Reanalysis and ultimately of GloFAS forecasts, which translate into better preparedness against large scale flooding and in the economic benefits of the related flood risk reduction (Pappenberger et al., 2015). Ongoing efforts and future research plans include an improved representation of the human influence to the global hydrological cycle through better estimates of the dominant water abstractions (e.g., irrigation, domestic, livestock, energy, manufacturing, mining, see Huang et al., 2018), to improve the estimation of flood warning thresholds for different forecast ranges (Alfieri et al., 2019), and to increase the use of remote sensing products for near real time updating of variables affecting the hydrological cycle, such as the Leaf Area Index (LAI) and surface water extent.

## 6 Data availability

The ERA5 dataset is distributed through the C3S Climate Data Store (https://cds.climate.copernicus.eu). Lisflood is an open source hydrological model and can be downloaded from the Github page https://ec-jrc.github.io/lisflood/, together with the calibration tool used in this work. Observed discharges are collected from around 30 providers, including regional and national hydro-meteorological institutes and international organizations. The largest number of station data was provided by the Global Runoff Data Centre (GRDC, https://www.bafg.de/GRDC/). The GloFAS- Reanalysis dataset v3.0 is freely available for download through the JRC Data Catalogue (https://data.jrc.ec.europa.eu/collection/id-00288) and near real time updates will be made available through the Copernicus Climate Data Store (https://cds.climate.copernicus.eu) once the operational version is released.

## CRediT authorship contribution statement

**Lorenzo Alfieri:** Conceptualization, Methodology, Formal analysis, Data curation, Writing - original draft, Writing - review & editing. **Valerio Lorini:** Formal analysis, Software, Data curation, Writing - review & editing. **Feyera A. Hirpa:** Writing - review & editing. **Shaun Harrigan:** Writing - review & editing. **Ervin Zsoter:** . **Christel Prudhomme:** Writing - review & editing. **Peter Salamon:** Writing - review & editing, Funding acquisition, Supervision.

## Declaration of Competing Interest

The authors declare that they have no known competing financial interests or personal relationships that could have appeared to influence the work reported in this paper.

## Footnotes

1 https://emergency.copernicus.eu

2 https://cds.climate.copernicus.eu
