# Supplementary material for: A global streamflow reanalysis for 1980–2018
Source: J Hydrol X. 2020 Jan;6:100049. doi: 10.1016/j.hydroa.2019.100049 (PMC6988497; doi:10.1016/j.hydroa.2019.100049)
Supplement: Supplementary data 2 [file mmc2.pdf]

Supplementary Information

A global streamflow reanalysis for 1980-2018

Lorenzo Alfieri[a*], Valerio Lorini [a], Feyera A. Hirpa[b], Shaun Harrigan[c], Ervin Zsoter[c], Christel Prudhomme[c,d,e], and Peter Salamon[a]

[a] European Commission, Joint Research Centre (JRC), Ispra, Italy

[b] School of Geography and Environment, University of Oxford, Oxford, UK

[c] European Centre for Medium-Range Weather Forecasts (ECMWF), Reading, UK

[d] Centre for Ecology and Hydrology, Wallingford, UK

[e] Geography Department, Loughborough University, Loughborough, UK

[*]Corresponding author: Lorenzo.Alfieri@ec.europa.eu

Contents of the Supplementary Information

- Figure S1
- Figure S2
- Figure S3
- Description of the reservoir routine in Lisflood
- Calibration_stations_Supplement.xlsx (file attached)

This table includes the list of 1226 calibration stations described in the article, together with a number of performance metrics in calibration and in validation.

Column legend:

ID	Station ID
Provider	Provider (acronym)
Station_name	Station name
River_Name	River name
River_Basin	River basin name
Country	2-digit ISO country code
Country_Name	Country name

Continent	Continent name
Drainage_Area_Provided	Drainage area provided [km^2]
Drainage_area_GloFAS	Drainage area in the GloFAS river network [km^2]
latitude	Latitude
longitude	Longitude
lat_GloFAS	Latitude of the station shifted to the GloFAS river network
long_GloFAS	Longitude of the station shifted to the GloFAS river network
Lake_ID	ID of the lake upstream the station
Reservoir_ID	ID of the reservoir upstream the station
Validation_Start	Start date of the validation period
Validation_End	End date of the validation period
Calibration_Start	Start date of the calibration period
Calibration_End	End date of the calibration period
KGE_cal	Kling-Gupta Efficiency in calibration
KGE_val	Kling-Gupta Efficiency in validation
NSE_cal	Nash-Sutcliffe Efficiency in calibration
NSE_val	Nash-Sutcliffe Efficiency in validation
R_cal	Pearson correlation coefficient in calibration
R_val	Pearson correlation coefficient in validation
PB_cal	Percent Bias in calibration [%]
PB_val	Percent Bias in validation [%]
RMSE_cal	Root Mean Square Error in calibration [m^3/s]
RMSE_val	Root Mean Square Error in validation [m^3/s]
MAE_cal	Mean Absolute Error in calibration [m^3/s]
MAE_val	Mean Absolute Error in validation [m^3/s]

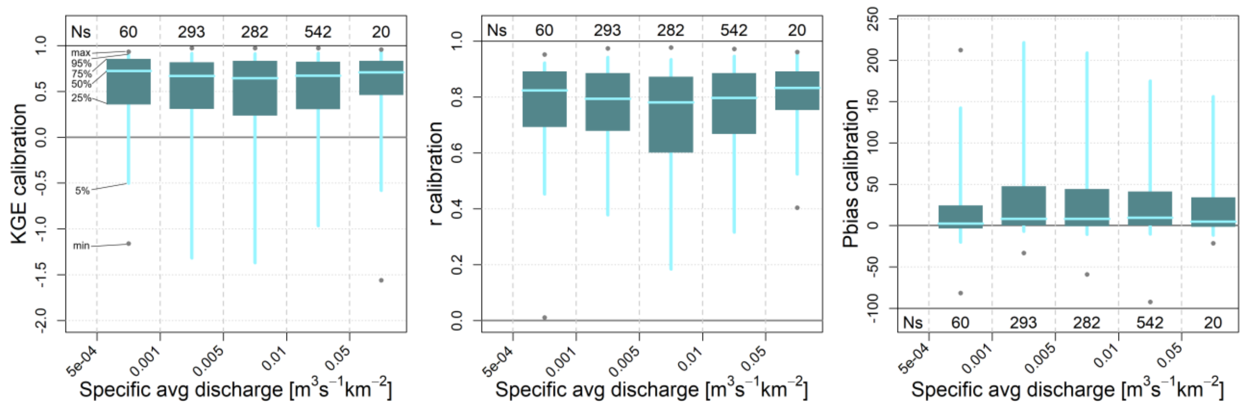

Figure S1: Box plot of the calibration performance by specific average discharge: KGE (left), correlation (center), and percent bias (right). Ns indicates the number of stations for each bin.

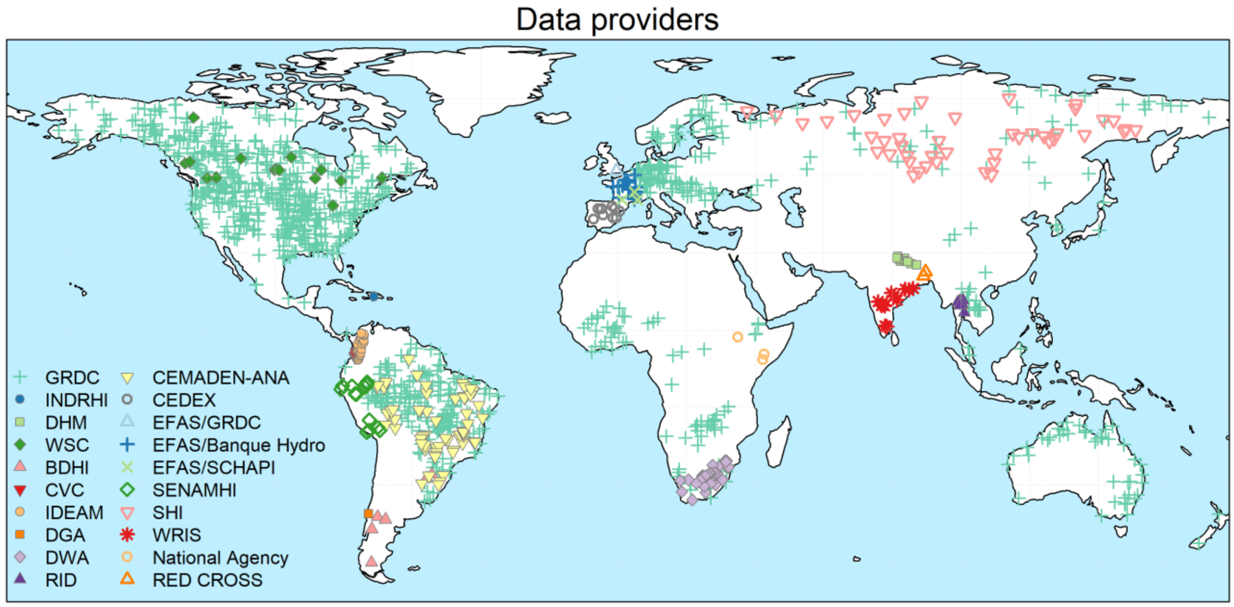

Figure S2: Data providers of the 1226 calibration stations.

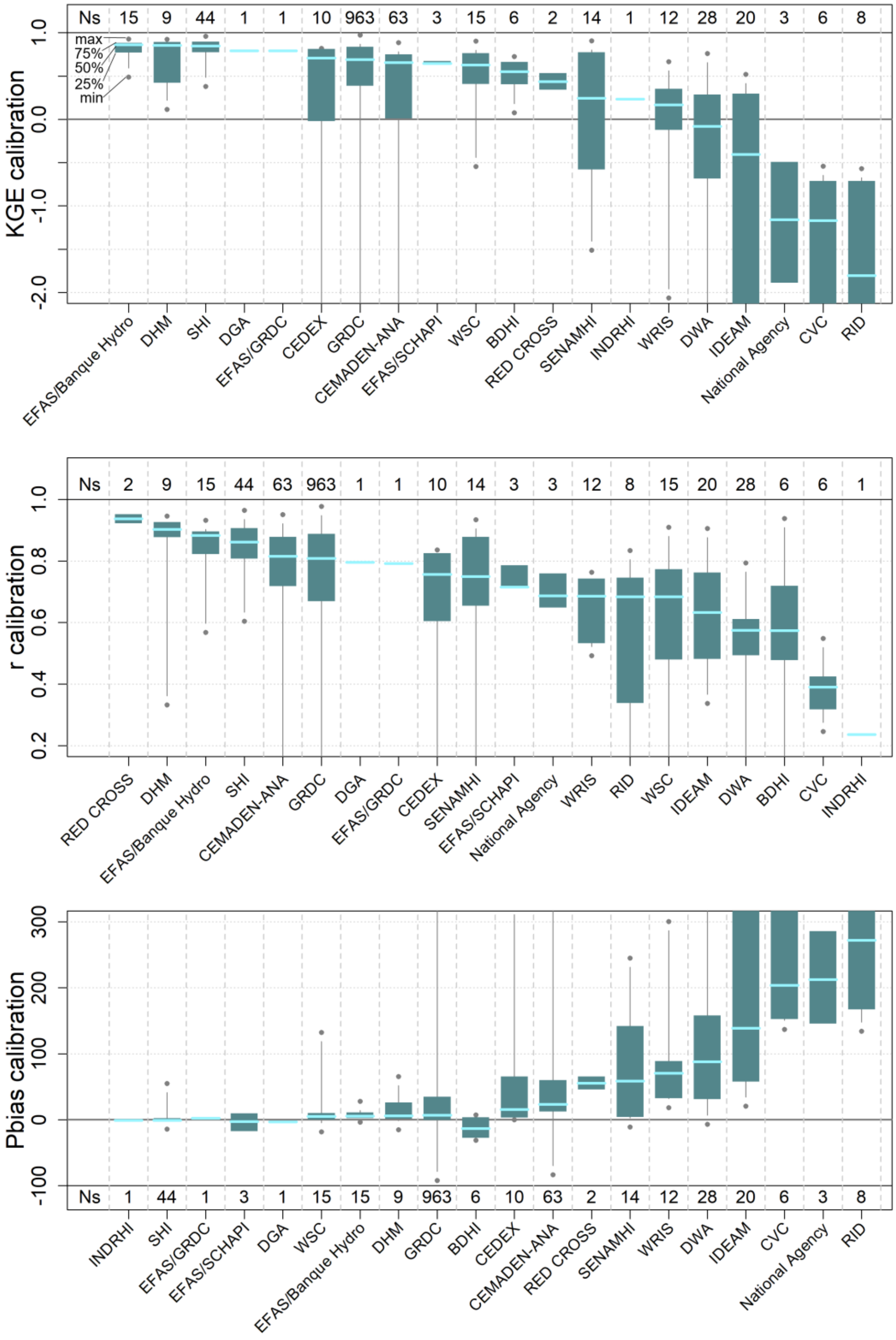

Figure S3: Box plot of the calibration performance by data provider: KGE (top), correlation (center), and percent bias (bottom). Ns indicates the number of stations for each bin. For each performance score, bins are ranked left to right from the most to the least skillful, according to the median of each bin.

Description of the reservoir routine in Lisflood

In Lisflood, reservoirs are simulated as point features in the channel network. The inflow into each reservoir equals the channel flow upstream the reservoir. The outflow is described by a set of rules. First, each reservoir has a total storage capacity *S* [m^3]. The relative filling of a reservoir, *F* , is a fraction between 0 and 1. There are three key values for filling levels.

- *Lc*: 'conservative storage limit'. This is the lower limit, which is larger than 0 as reservoirs are never completely empty.
- *Lf* : 'flood storage limit'. This is the upper limit, which is smaller than 1 as reservoirs are never filled completely for safety reasons.
- *Ln*: is the available capacity of a reservoir between *Lf* and *Lc*.

Three additional parameters are used to define the regulation of the reservoir outflow:

- 'minimum outflow' (*Omin*, [m^3/s]) which is maintained for e.g. ecological reasons;
- 'non-damaging outflow' (*Ond*, [m^3/s]) is the maximum possible outflow that will not cause problems downstream; and
- 'normal outflow' (*Onorm*, [m^3/s]) is the outflow used when the reservoir is within its 'normal storage' filling level.

Depending on the relative filling of the reservoir, the outflow (*Ores*, [m^3/s]) is calculated as:

If $F \leq 2 \cdot Lc$, then:

$$Ores = min(Omin, F \cdot S \cdot 1/\Delta t)$$

If $Ln \geq F > 2Lc$, then:

$$Ores = Omin + (Onorm - Omin) \cdot (F - 2Lc)/(Ln - 2Lc)$$

If $Lf \geq F > Ln$, then:

$$Ores = Onorm + ((F - Ln)/(Lf - Ln)) \cdot \max((Ires - Onorm),(Ond - Onorm))$$

If $F > Lf$, then:

$$Ores = \max(Ond, S(F - Lf)/\Delta t)$$

where:
S: Reservoir storage capacity [m^3]
F: Reservoir fill (fraction, 1 at total storage capacity) [-]
Lc: Conservative storage limit [-]
Ln: Normal storage limit [-]
Lf: Flood storage limit [-]
Omin: Minimum outflow [m^3/s]
Onorm: Normal outflow [m^3/s]
Ond: Non-damaging outflow [m^3/s]
Ires: Reservoir inflow [m^3/s]
